# Low proportion of follicular regulatory T cell in renal transplant patients with chronic antibody-mediated rejection

**DOI:** 10.1038/s41598-017-01625-3

**Published:** 2017-05-02

**Authors:** Wen Chen, Jian Bai, Haiyan Huang, Lili Bi, Xiangrui Kong, Yu Gao, Yong Han, Li Xiao, Bingyi Shi

**Affiliations:** Beijing Key Laboratory of Immunology Regulatory and Organ Transplantation, Basic Research Lab of Organ Transplant Institute, 309th Hospital of the People’s Liberation Army, Beijing, 100091 China

## Abstract

Follicular regulatory T (Tfr) cell can effectively regulate humoral immunity, but its function and mechanism in antibody-mediated rejection (AMR) after organ transplantation remains unclear. Here we detected follicular helper T (Tfh) cell subsets in 88 renal transplant patients with chronic renal allograft dysfunction (40 with AMR and 48 without AMR). The ratio of Tfr cells in renal graft tissues and peripheral blood of AMR patients significantly decreased, while the ratio of IL-21-producing Tfh cells (Tfh2 and Tfh17) significantly increased, compared to non-AMR patients. When tested in functional assays, Tfr cells from both AMR and non-AMR patients exerted equivalent inhibitory function. Tfr cell transplantation or CTLA-4 virus transfection could significantly inhibit IL-21 secretion from Tfh cells of these patients, further suppress the proliferation and differentiation of B cells. CTLA-4 blocking, IL-10 and TGF-β neutralization could partially weaken such inhibitory effect of Tfr cells. Besides, our study found that sirolimus reduced the ratio of Tfr cells, while cyclosporine and tacrolimus had no significant effect on Tfr cells. In a word, renal transplant patients with AMR have low proportion of Tfr cells but these cell exerted normal function.

## Introduction

Over these years, the advances of immunosuppressive therapy have significantly reduced the incidence rate of T cell-mediated rejection after renal transplantation and substantially increased the short-term survival rate of renal graft, but the long-term prognosis is unsatisfactory^1, 2^. Antibody-mediated rejection (AMR) gradually becomes the most critical cause to the occurrence of dysfunction in the late period of renal graft and there is no available clinical prevention and treatment measure^3^. Follicular helper T cell (Tfh cell) plays a crucial role in the generation and development of AMR, which helps B cells differentiation into plasma cells and the production of donor-specific antibody (DSA) through the secretion of IL-21^4, 5^. The number of Tfh cells in patient is stable before or after renal transplantation, but the capacity of Tfh cells for IL-21 secretion significantly reduces after renal transplantation, which indicates that immunosuppressive therapy may impact the function and phenotypic change of Tfh cells^6^.

Tfh cell subsets are plastic, which may transform among each other under a specific microenvironment^7^. Tfh cells can be transformed from Th1, Th2 and Th17 cells and the transformed cells still partially keep the cell capacity before transformation^8^. For example, Tfh cells sourced from Th1 (Tfh1 cells) can secrete IFN-γ, Tfh cells sourced from Th2 (Tfh2 cells) can secrete IL-4, IL-5 and IL-13, Tfh cells sourced from Th17 (Tfh17 cells) can secrete IL-17 and IL-22, while only Tfh2 cells and Tfh17 cells can secrete IL-21 and help the proliferation and differentiation of B cells^9^.

Recent studies have discovered that a follicular regulatory T cell (Tfr cell) exists in organism, which has the function of inhibiting the formation of germinal center and the differentiation of B cells^10–12^. However, the mechanism of Tfr cells inhibiting humoral immunity remains unclear, relevant studies suggest that Tfr cell is sourced from the precursor cell of Treg and its biological function can be fulfilled through CTLA-4 or the production of inhibitory cytokines (IL-10 and TGF-β)^13–15^. To our knowledge, the relationship between Tfr cells and rejection has not been reported yet. The research on the relationship between Tfh cells, Tfr cells and AMR may offer a new route to the effective prevention and correction of AMR and the promotion for the long-term survival of graft.

## Results

### Patients

Our study sample included 128 recipients and all patients received similar induction therapy with tacrolimus, mycophenolate mofetil and prednisone acetate. There were no significant differences in the total dosages of immunosuppressive agents. Baseline data were shown in Table 1 and Table S1. Patients with renal transplantation were studied at a average time of 4.77 years. Eighty-eight of 128 patients with renal transplantation were diagnosed as chronic renal allograft dysfunction (CRAD) by transplant physicians and their creatinine value was 235.3 ± 48 umol/L. Within the group of patients with CRAD, 40 had been diagnosed as AMR, as both positive DSA detection in serum and positive C4d staining in allograft.

**Table 1:** The baseline and clinical characteristics of CRAD patients in renal transplantation.

	Total (n = 88)	AMR (n = 40)	Non-AMR (n = 48)	*P* value
Age (yr)	41.7 ± 10.7	44.7 ± 12.0	40.4 ± 10.3	0.431
Male gender	28	28	14	0.346
BMI (Kg/m^2^)	25.7 ± 5.3	26.3 ± 4.3	24.4 ± 5.1	0.132
Time after transplantation	4.86 ± 1.73	5.57 ± 1.27	4.78 ± 1.99	0.134
White blood cell	7.8 ± 3.3	7.2 ± 2.3	8.1 ± 3.7	0.481
Lymphocyte	26.7 ± 8.2	28.6 ± 12.1	25.8 ± 8.0	0.594
Tfh cell	1.28 ± 0.78	1.32 ± ± 0.71	1.24 ± 0.83	0.685
Tfh1 cell	0.4 ± 0.07	0.36 ± 0.05	0.44 ± 0.08	0.054
Tfh2 cell	0.39 ± 0.057	0.42 ± 0.064	0.36 ± 0.051	0.023
Tfh17 cell	0.36 ± 0.034	0.46 ± 0.041	0.28 ± 0.029	<0.01
Tfr cell	0.11 ± 0.013	0.08 ± 0.01	0.14 ± 0.016	0.016
Monocyte	0.61 ± 0.3	0.57 ± 0.2	0.63 ± 0.3	0.576
Urea nitrogen	8.11 ± 4.5	10.4 ± 9.3	7.1 ± 2.5	0.257
Creatinine (umol/L)	135.3 ± 48	266.1 ± 71.8	221.8 ± 27	0.153
Uric acid	396.6 ± 73	390.6 ± 111.5	399.2 ± 69	0.821
Total protein	68.5 ± 10.6	73.5 ± 20.2	66.4 ± 11.1	0.104
GFR	56.9 ± 16.2	46.9 ± 24.1	61.3 ± 11.3	0.14
Triglyceride	1.77 ± 0.95	1.71 ± 1.36	1.80 ± 1.0	0.826
Total cholesterol	4.77 ± 1.4	4.80 ± 1.3	4.76 ± 1.53	0.942

### Changes of Tfh cell subsets in AMR

**Figure 1:**
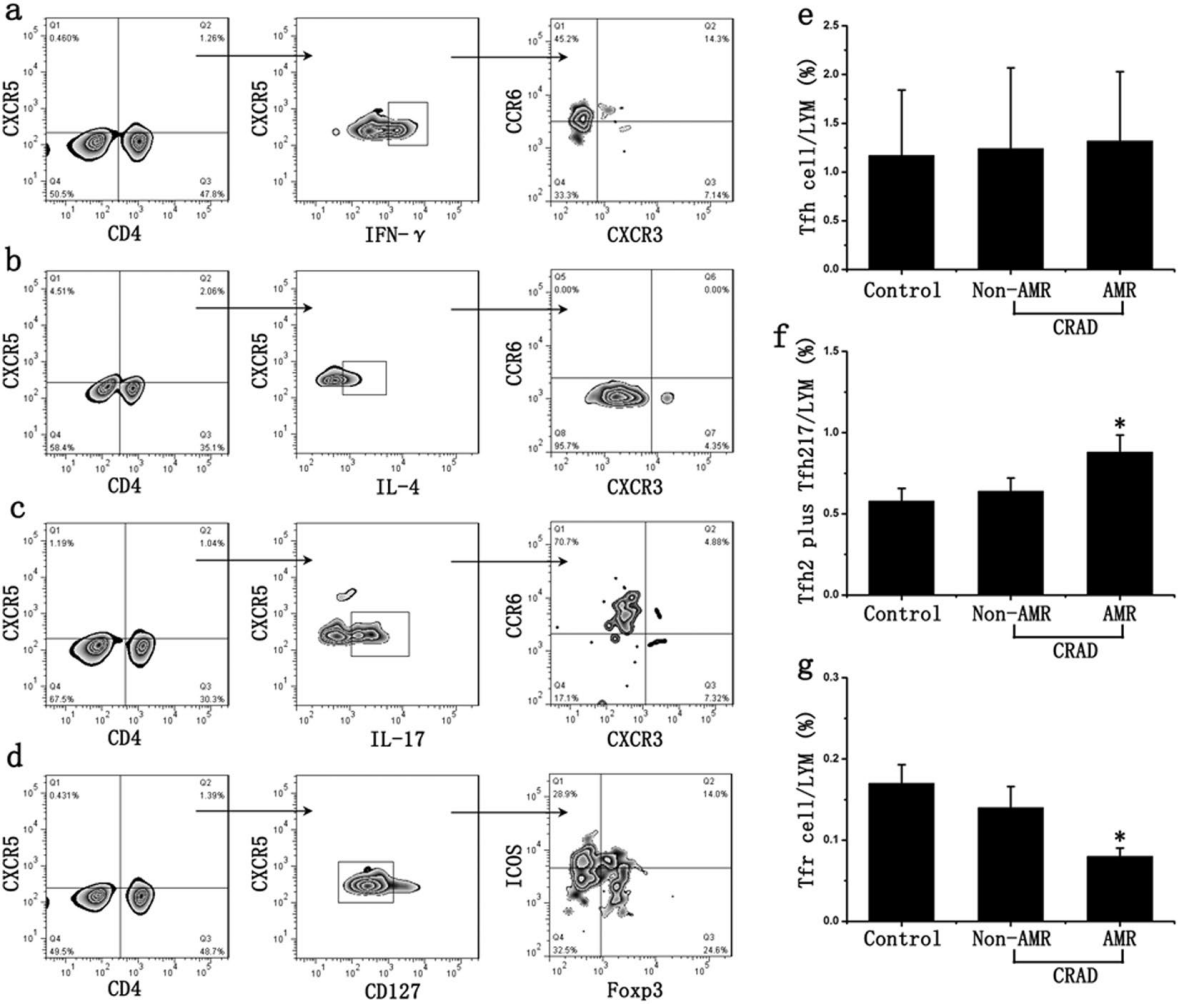
Changes of Tfh cell subsets in AMR. (**a**) CD4, CXCR5, IFN-γ, CXCR3 and CCR6 antibodies were used to detect Tfh1 cells; (**b**) CD4, CXCR5, IL-4, CXCR3 and CCR6 antibodies were used to detect Tfh2 cells; (**c**) CD4, CXCR5, IL-17, CXCR3 and CCR6 antibodies were used to detect Tfh17 cells; (**d**) CD4, CXCR5, ICOS, Foxp3 and CD127 antibodies were used to detect Tfh and Tfr cells; (**e**) CD4+CXCR5+ICOS+Tfh cell in lymphocyte (LYM) had no significant difference between AMR group and non-AMR group; (**f**) The ratio of IL-21-producing Tfh cells (CD4+CXCR5+IL-4+ CXCR3-CCR6-Tfh2 and CD4+CXCR5+IL-17+ CXCR3−CCR6+Tfh17 cell) in AMR group was significantly higher than that of non-AMR group; (**f**) The ratio of CD4+CXCR5+ICOS+FOXP3+CD127−Tfr cells in AMR group was significantly lower than that of non-AMR group. Two-sided Wilcoxon rank sum test: *p < 0.05 (n = 40) *versus* non-AMR. Values are mean ± SD.

Flow cytometry results indicated that the ratio of Tfh (CD4+CXCR5+ICOS+) in peripheral blood of control and CRAD patients had no significant difference, while the ratio of Tfh17 (CD4+CXCR5+IL-17+CXCR3−CCR6+) and Tfh2 (CD4+CXCR5+IL-4+CXCR3−CCR6−) cells in peripheral blood of CRAD patients was significantly higher than that of control patients. CRAD patients were further divided into AMR group and non-AMR group and the results showed that creatinine levels, Tfh and Tfh1(CD4+CXCR5+IFN-γ+CXCR3+CCR6−) cells had no significant difference between these two groups, but the ratio of IL-21-producing Tfh cells (Tfh2 and Tfh17 cells) in AMR group was significantly higher than that of non-AMR group, and the ratio of Tfr (CD4+CXCR5+ICOS+FOXP3+CD127−) cells was significantly lower than that of non-AMR group (Fig. 1).

Then we analyzed the correlation between clinical data. There is no significant correlation between DSA and percentages of circulating Tfh cells (r = 0.1596, p = 0.3911). We further analyzed Tfh subset, a significant inverse correlation was showed between DSA and percentages of circulating Tfr cells (r = −0.5090, p = 0.0035). Percentages of circulating IL-21-producing Tfh cells (Tfh2 and Tfh17) were positive correlated with DSA (r = 0.6124, p = 0.0003). However, there were no correlations between Tfh cells and creatinine values (r = −0.01916, p = 0.9185), Tfr cells and creatinine values (r = −0.2311, p = 0.211). These results showed that reduced Tfr cells and increased IL-21-producing Tfh cells (Tfh2 and Tfh17 cells) in renal transplantation correlates with occurrence of AMR (Table S2).

### Decreased frequency of Tfr cells associated with AMR in renal graft tissues

**Figure 2:**
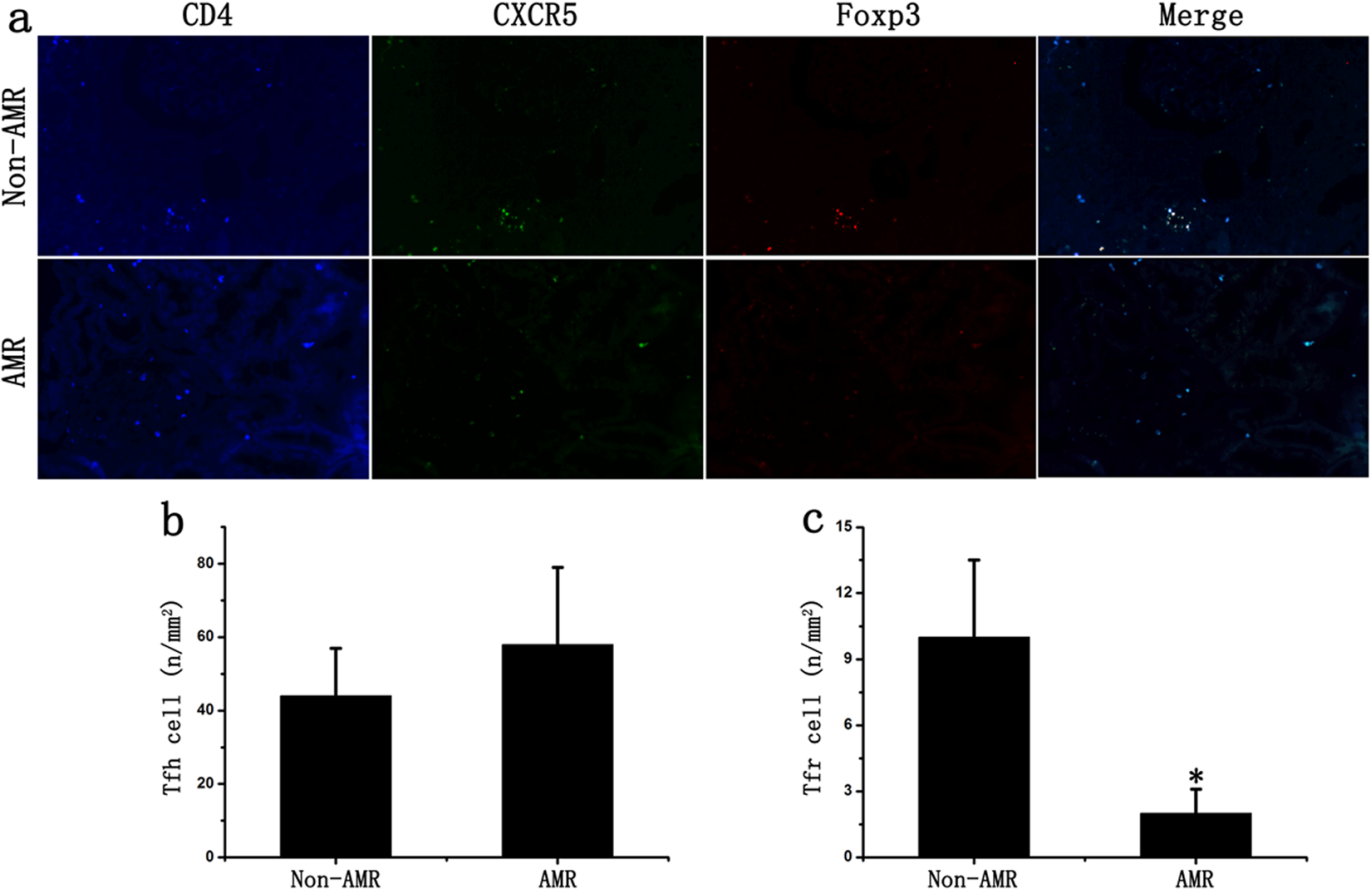
Changes of Tfr cells in renal graft tissues. (**a**) The number of Tfh cells and Tfr cells in renal graft tissues of CRAD patients was detected using immunofluorescence staining. (**b**) There is no statistical difference with the number of Tfh cells between AMR group and non-AMR group. (**c**) The ratio of Tfr cells in renal graft tissues of AMR group was significantly lower than that of non-AMR group. Two-sided Wilcoxon rank sum test: *p < 0.05 (n = 10) *versus* Non-AMR. Values are mean ± SD.

The number of Tfh cells (CD4+CXCR5+ cells) and Tfr cells (CD4+CXCR5+ Foxp3+ cells) in renal graft tissues of CRAD patients was detected using immunofluorescence method. Results showed that no statistical difference with respect to the number of Tfh cells was identified between AMR group and non-AMR group, while the number of Tfr cells in renal graft tissues of AMR patients was significantly lower than that of Non-AMR patients (Fig. 2).

### Effects of anti-rejection drugs on Tfh cell subsets

**Figure 3:**
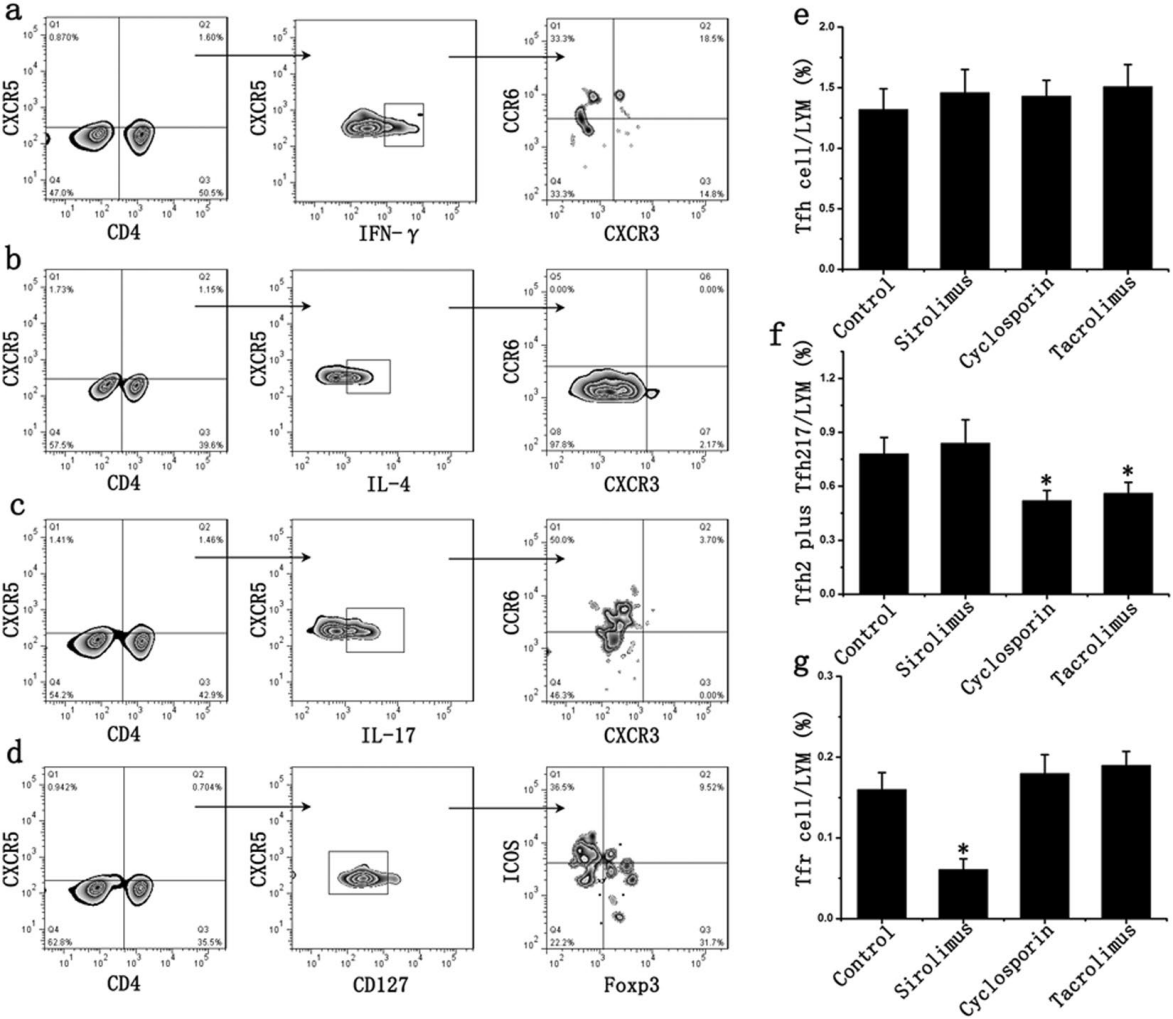
Effects of anti-rejection drugs on Tfh cell subsets. (**a**) CD4, CXCR5, IFN-γ, CXCR3 and CCR6 antibodies were used to detect Tfh1 cells; (**b**) CD4, CXCR5, IL-4, CXCR3 and CCR6 antibodies were used to detect Tfh2 cells; (**c**) CD4, CXCR5, IL-17, CXCR3 and CCR6 antibodies were used to detect Tfh17 cells; (**d**) CD4, CXCR5, ICOS, Foxp3 and CD127 antibodies were used to detect Tfh and Tfr cells; (**e**) The anti-rejection drugs had no significant effect on the proportion of Tfh cells in lymphocytes (LYM). (**f**) Cyclosporine and tacrolimus reduced the proportion of IL-21-producing Tfh cells (Tfh2 and Tfh17 cells) in lymphocytes. (**g**) Sirolimus could reduce the proportion of Tfr cells in lymphocytes after being stimulated for 48 hours. Student’s t test: *p < 0.05 (n = 6) *versus* Control. Values are mean ± SD.

Flow cytometry results showed that all anti-rejection drugs had no significant effect on the number of Tfh cells. The effects of these drugs on Tfh cell subsets were further examined and results indicated that sirolimus could reduce the ratio of Tfr cells after being stimulated for 48 hours, which has no significant effect on Tfh1, Tfh2 and Tfh17 cells; cyclosporine and tacrolimus can increase the ratio of Tfh1 cells and reduce the ratio of IL-21-producing Tfh cells (Tfh2 and Tfh17 cells), which has no significant effect on Tfr cells (Fig. 3).

Tfh cells were then sorted and used for mixed lymphocyte culture (MLC). Results showed that the concentration of IL-21 significantly increased in sirolimus group if compared to the control group; in cyclosporine and tacrolimus group, the concentration of IL-21 decreased, but no significant difference with respect to the ratio of B cells was identified if compared to the control group (Figures S1 and S2).

### Tfr cells inhibited B cell proliferation and differentiation in renal transplantation

**Figure 4:**
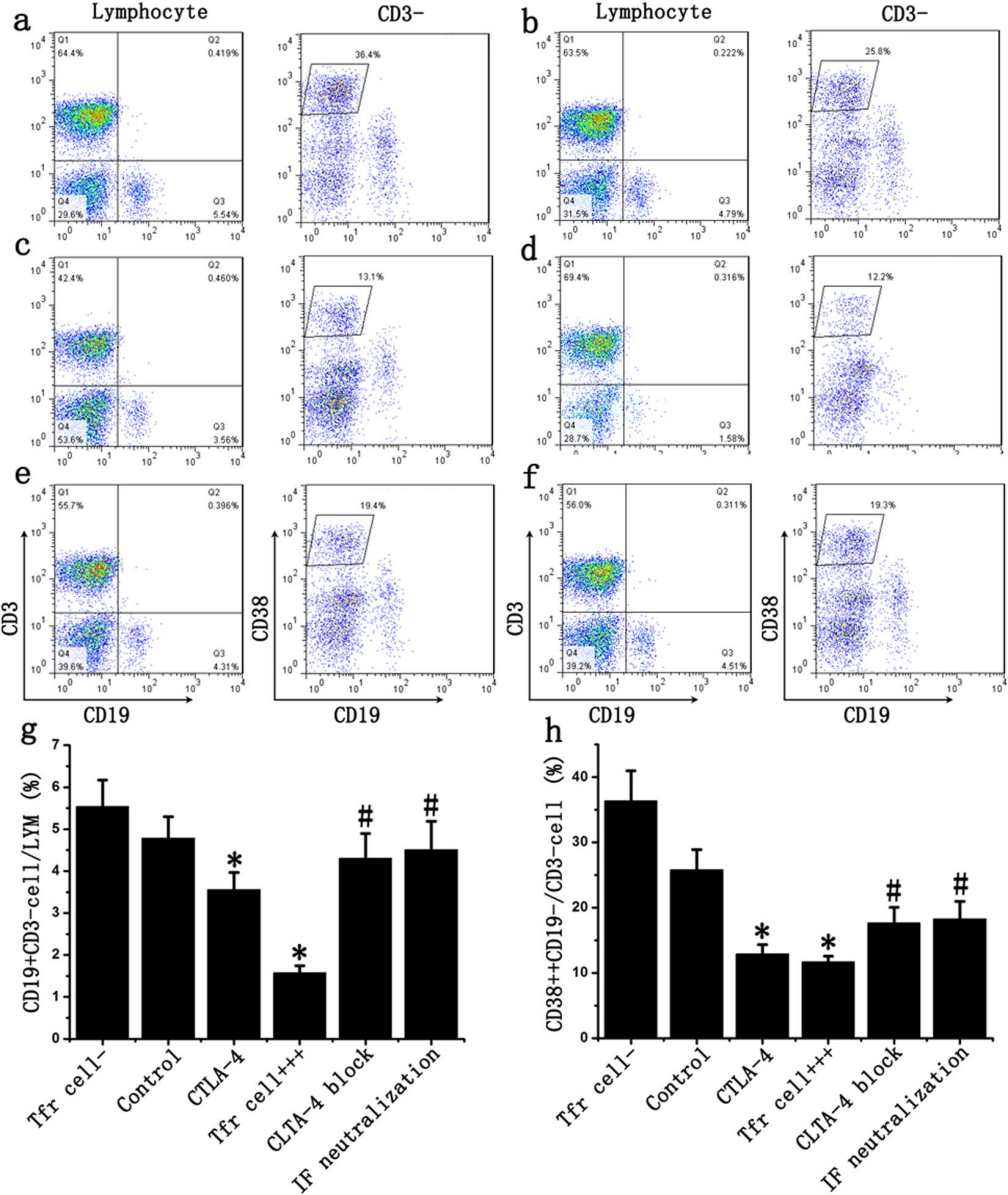
Tfr cells inhibited B cell to differentiate into plasma cells in renal transplantation. Tfr cells were sorted from AMR patients and divided into many groups: Tfr cells deleted group (Tfr cell−, **a**), Tfr cells normal ratio group (Control, **b**), CTLA-4 group (**c**) and Tfr cells ratio increase group (Tfr cell+++ **d**). In Tfr cell+++ group, CTLA-4 antibody was used to block CTLA-4 of Tfr cells (CTLA-4 block (**e**); IL-10 and TGF-β antibody was used to neutralize the inhibitory factors from Tfr cells (IF neutralization, **f**). (**g**) The ratio of B cells in Tfr cells+++ and CTLA-4 group decreased compared with control group, while CTLA-4 blocking, IL-10 and TGF-β neutralization could partially suppressed this decrease of B cells. (**h**) Tfr cells and CTLA-4 inhibit B cell differentiation into plasma cells and CTLA-4 blocking, IL-10 and TGF-β neutralization could partially lessen the inhibitory effect of Tfr cells. Student’s t test: *p < 0.05 (n = 6) *versus* Control; ^#^p < 0.05 (n = 6) versus Tfr cells+++. Values are mean ± SD.

Results indicated that B cells in Tfr cells deleted group significantly proliferated and differentiated to plasma cells, with an increase of 15.7%, 41.1% respectively if compared to the control group; the ratio of B cells and plasma cells in Tfr cells+++ group decreased by 67% and 52.7% respectively; CTLA-4 blocking, IL-10 and TGF-β neutralization could partially lessen the inhibitory effect of Tfr cells, which led to the ratio of B cells and plasma cells increased by 161%, 59% and 185%, 63% respectively. These results indicated that Tfr cells might be an effective target for the treatment of AMR after renal transplantation (Fig. 4). ELISA results showed that Tfr cells could inhibit the IgG and IgA production from plasma cells, but had no significant effect on IgM production (Figure S3).

**Figure 5:**
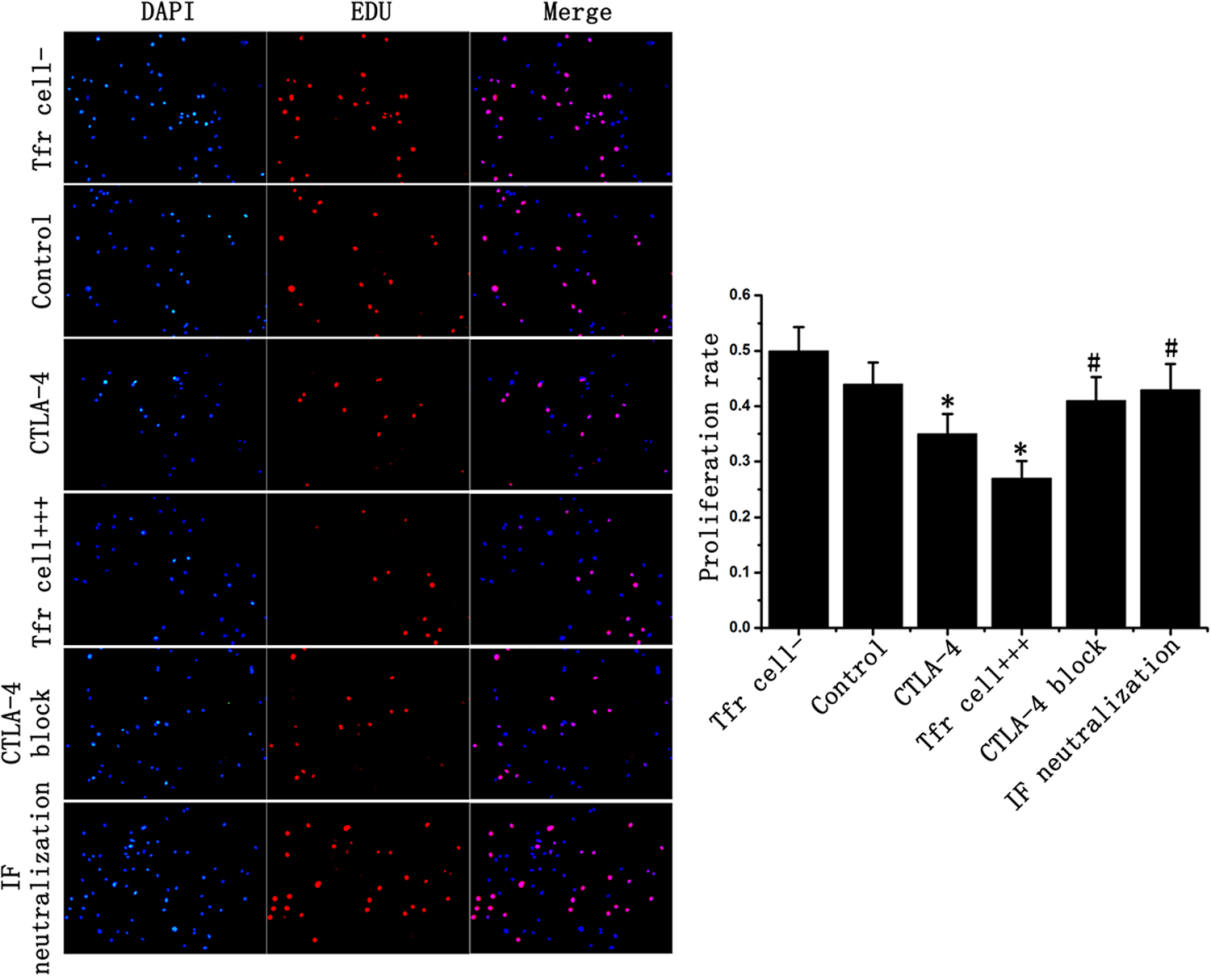
Tfr cells inhibit B cell proliferation in renal transplantation. After mixed lymphocyte culture, B cells were sorted and cultured for 48 hours. All B cells were stained with DAPI (blue) and proliferating cells were stained with EDU (Red). The results showed B cell proliferation rate in Tfr cells+++ and CTLA-4 group was significantly lower than that in the control group. CTLA-4 block or inhibitory factor could partly weakened this inhibitory effect of Tfr cell. Student’s t test: *p < 0.05 (n = 3) *versus* Control; ^#^p < 0.05 (n = 3) versus Tfr cells+++. Values are mean ± SD.

To further detect the effect of Tfr cells on B cell proliferation, we performed EDU experiments. The results showed that Tfr cells could markedly inhibit B cell proliferation, with an decrease of 45.8% if compared to the control group. CTLA-4 blocking, IL-10 and TGF-β neutralization could also partially weaken the inhibitory effect of Tfr cells, which increased the proliferation rate by 57.7% and 65.4% respectively, compared to the Tfr cell+++ group (Fig. 5).

### CTLA-4 over expression inhibited B cell differentiation

**Figure 6:**
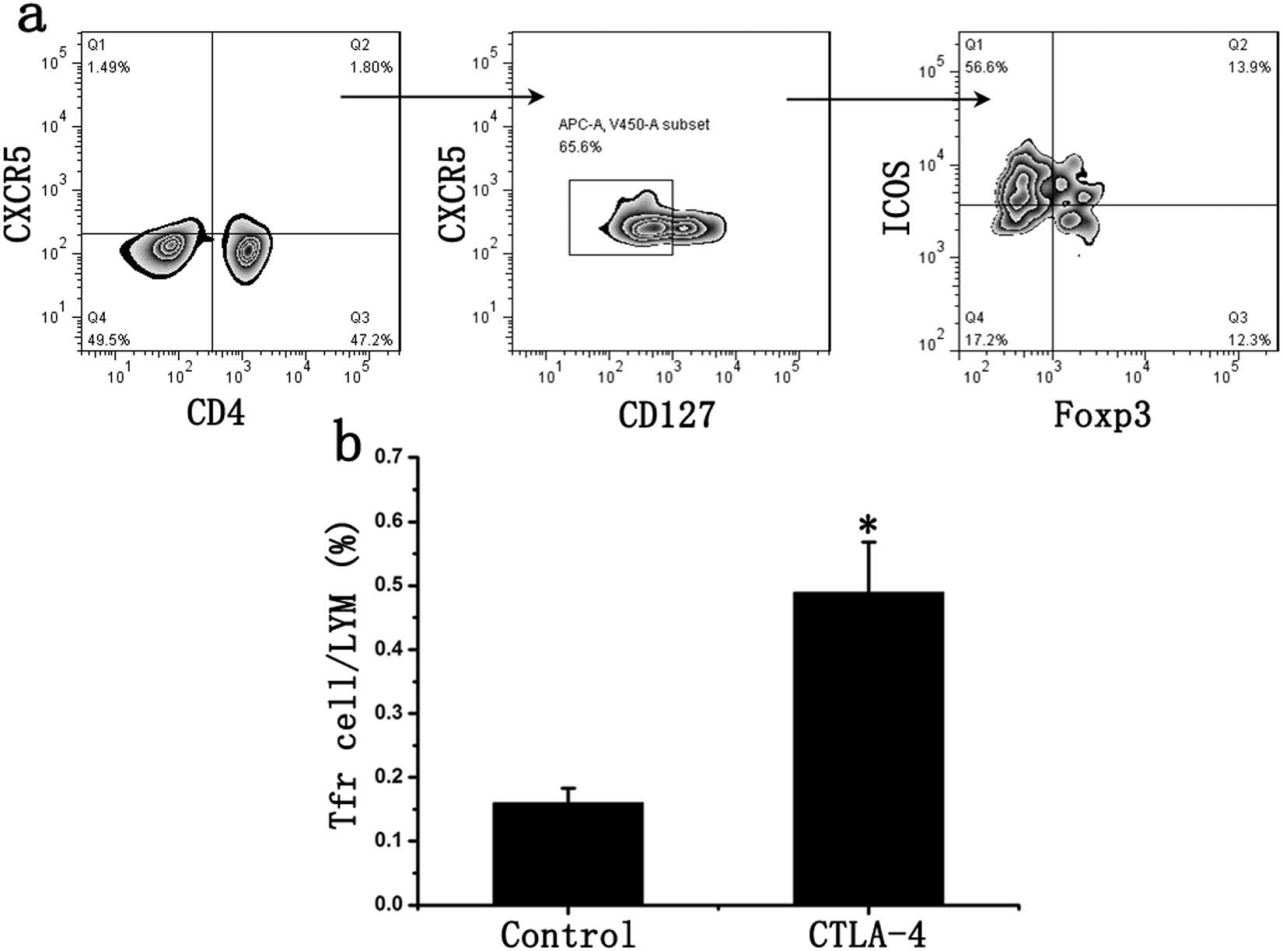
CTLA-4 increased Tfr cell proportion in lymphocyte. (**a**) CD4, CXCR5, ICOS, Foxp3 and CD127 antibodies were used to detect Tfh and Tfr cells; (**b**) CTLA-4 could significantly increase the ratio of Tfr cells in Tfh cells. Student’s t test: *p < 0.05 (n = 6) *versus* Control. Values are mean ± SD.

It has been proved that CTLA-4 could significantly increase the ratio of Tfr cells^16^. Our results also showed that Tfr cell ratio increased by 171% 48 hours after transfection (Fig. 6). Then MLC was carried out and the ratio of B cells and plasma cells in CTLA-4 group significantly decreased by 25.7% and 49.2% respectively, if compared to the control group (Fig. 4c).

### Tfr cells in AMR patients exerted normal inhibitory function

**Figure 7:**
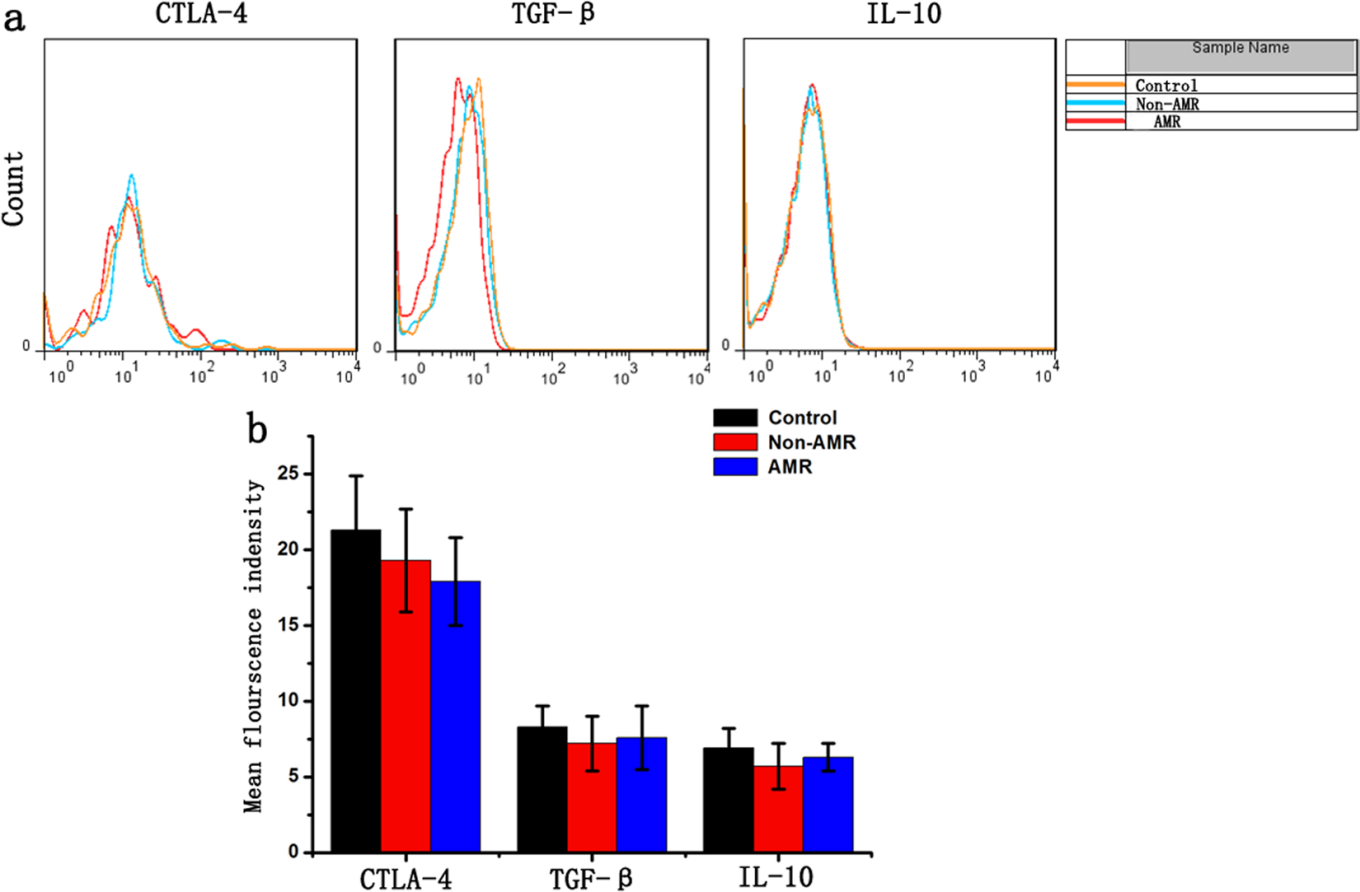
Tfr cells in AMR patients exerted normal inhibitory function. (**a**) Tfr cells were isolated from patients in each group and detected the expression of IL-10, TGF-β and CTLA-4; (**b**) CTLA-4, IL-10 and TGF-β expression of Tfr cells isolated from AMR patients was similar to that from control patients or CRAD patients without AMR.

The previous experiments showed a correlation between the number of Tfr cell and their inhibitory effect. Next we isolated Tfr cells from patients in each group and detected the expression of IL-10, TGF-β and CTLA-4. The results showed no significant differences between control and CRAD patients. Among CRAD patients, we also could not found the significant differences between AMR and Non-AMR patients (Fig. 7). We further performed B cell and Tfr cell co-culture experiments and the results showed Tfr cells isolated from AMR patients exerted the similar inhibitory effect on B cell proliferation and differentiation into plasma cells (Figure S4). These results indicated that Tfr cells isolated from AMR patients exerted the similar inhibitory effect as those cells from CRAD patients without AMR or control patients.

## Discussion

AMR is the most critical factor that affects the long-term survival of renal graft, but no effective prevention and treatment is available^17^. Tfh cells play a key effect in the occurrence and development of AMR, Tfh2 and Tfh17 cells can secrete a large quantity of IL-21, facilitate B cells proliferation and differentiation into plasma cells and the production of DSA, Tfr cells have an inhibitory effect on the differentiation of B cells^18, 19^. Our study revealed that the ratio of Tfr cells in peripheral blood and renal graft tissues of AMR patient was significantly lower than that of Non-AMR patient, while the ratio of Tfh2 and Tfh17 cells significantly increased. Decreased Tfr cells and increased IL-21-producing Tfh cells (Tfh2 and Tfh17) in transplant patients are polarized to the direction of facilitating the secretion of IL-21 and promote B cells proliferation and differentiation into plasma cells, which would lead to the occurrence of AMR. Correlation analysis results confirmed reduced Tfr cells and increased IL-21-producing Tfh cells (Tfh2 and Tfh17) in renal transplantation correlates with occurrence of AMR.

Clinically, patients after organ transplantation need to administer a lot of anti-rejection drugs to inhibit the T cells-mediated rejection^20, 21^, but the effects of immunosuppressive microenvironment after organ transplantation on changes of Tfh cell subsets and production of DSA were scarcely reported. de Graav found that the number of Tfh cells didn’t significantly change, but the secretion of IL-21 significantly decreased, after the immunosuppressive therapy using tacrolimus and mycophenolate mofetil^6^. Our study revealed that tacrolimus can promote the increased ratio of Tfh1 cells with no ability of secreting IL-21, and decrease the ratio of IL-21 producing Tfh cells, which would result in the decreased total secretion of IL-21. Croze *et al*. summarized 270 patients after renal transplantation and found that the production of DSA and the occurrence of AMR significantly increased after converting the calcineurin inhibitor (cyclosporine) to mTOR pathway inhibitor (rapamycin), which can be properly explained by our experiment results^22^. Cyclosporine can facilitate the increase of Tfh1 cells, inhibit IL-21 producing Tfh cells and cause the decreased secretion of IL-21; however, the inhibitory effect of cyclosporine and tacrolimus on production of IL-21 is limited, and the staphylococcal enterotoxin B (SEB)-mediated proliferation and differentiation of B cells cannot be effectively reversed in co-culture experiment.

Tfr cell is a regulatory follicular helper T cell that has been recently discovered, with the functions such as inhibiting the formation of germinal center and the differentiation of B cells, which is considered as an effective target for the treatment of AMR after organ transplantation^10, 13, 15, 23^. Our study is the first to affirm that Tfr cell transplantation may effectively inhibit the occurrence of AMR after renal transplantation, and it is believed that such mechanism may directly or indirectly function: directly contact plasma cells and have an inhibitory effect; or regulate the secretion of IL-21 from Tfh cells^16, 24–26^. Our results indicate that the role of Tfr cells is multi-channel that impacts the secretion of IL-21 as well as the proliferation and differentiation of B cells through CTLA-4 or the secretion of inhibitory molecules, ultimately inhibiting the occurrence of AMR. CTLA-4 has the function of significantly facilitating the increased ratio of Tfr cells^16^. Results showed that CTLA-4 can significantly reduce the secretion of IL-21 and inhibit B cell differentiation into plasmocyte, which might become a new potential target for the prevention and treatment of AMR.

In conclusion, renal transplant patients with AMR have low proportion of Tfr cells but these cell exerted normal function. Tfr cell therapy might be an effective target for the prevention and treatment of AMR in organ transplantation.

## Materials and Methods

### Ethics statement

This study was approved by the Ethics Committee of 309th hospital of PLA and was performed according to the principles of the Declaration of Helsinki. The experiments involving human subjects were approved by Institutional Review Board of 309th hospital of PLA. All participants provided written informed consent, and the ethics committee approved the consent procedure.

### Collection of clinical samples

The patients with end-stage renal disease, having deceased renal transplantation at 309th hospital of PLA, were enrolled in our study. Exclusion criteria are the patients who required a combined transplantation or those less than 18 years old. In China, citizen’s voluntary donations have been the only channel of organ transplantation. All donor kidney were derived from voluntary donations and this study was approved by Institutional Review Board of 309th hospital of PLA.

### Detection of clinical samples

The peripheral blood of control, chronic renal allograft dysfunction (CRAD) with AMR, CRAD without AMR patients after renal transplantation was collected, mononuclear cells were separated by the lymphocytes separation medium (TBD), then labeled by the flow type antibodies CD4, CXCR5, Foxp3, ICOS, IL-17, IL-4, IFN-γ, CD127, CXCR3 and CCR6 (All purchased from BD), and sent to the flow cytometry after three washes using PBS.

Renal biopsies of all groups of patients as mentioned above were selected and cut into slices in 4 µm thickness after the formalin fixation and paraffin embedding. The primary antibodies CD4, CXCR5 and Foxp3 (All purchased from Abcam), were added and stay overnight at 4 °C. After three washes using PBS, fluorescent secondary antibody was added and incubated in 37 °C thermostat for 30 min, and observed under the fluorescent microscope after three washes using PBS. We randomly selected 7 horizons in each biopsy and counted positive cell number.

### Effects of anti-rejection drugs on Tfh cell subsets

The peripheral blood of healthy volunteers was obtained and mononuclear cells were separated using lymphocytes separation medium, after being cultured for 48 hours, cyclosporine (250 ng/ml), sirolimus (10 ng/ml) and tacrolimus (10 ng/ml) were used for stimulation respectively, then labeled by the flow type antibodies CD4, CXCR5, Foxp3, ICOS, IL-17, IL-4, IFN-γ, CD127, CXCR3, CCR6 and CTLA-4, and sent to the flow cytometry after three washes using PBS. And the supernatant of cell culture was collected and the content of IL-21 in the supernatant was detected using ELISA.

### Mixed lymphocyte culture (MLC)

After the lymphocytes collected from healthy volunteers were stimulated using cyclosporine, sirolimus or tacrolimus for 48 hours respectively, Tfh cells in peripheral blood were sorted using magnetic bead selection. SEB could promote T cell proliferation and secretion of many cytokine, so the sorted Tfh cells and lymphocyte (Tfh cell-) were used to co-cultured in the cell culture medium with 1 mg/ml SEB^9, 27^. After being cultured for 48 hours, the cells were labeled by the flow type antibodies CD3 and CD19, and sent to the flow cytometry after PBS washing.

### Effects of Tfr cells on B cells of AMR patients

The peripheral blood of AMR patients was obtained and lymphocyte were separated using lymphocytes separation medium, and Tfr cells were sorted through FCM sorting. In CTLA-4 group, lymphocytes were transfected with human CTLA-4 expression lentivirus and then replaced with the fresh medium 6 hours after transfection, and then sorted after being cultured for 48 hours. The experiment was divided into many groups: Tfr cells deleted group (Tfr cell−), Tfr cells normal ratio group (Control), Tfr cells ratio increase group (Tfr cell+++) and CTLA-4 group. Later, Tfr cells were co-cultured with lymphocytes (Tfr−) from the same patient in the cell culture medium. To further investigate the mechanism, CTLA-4 antibody was used to block CTLA-4 of Tfr cells; IL-10 and TGF-β antibody was used to neutralize the inhibitory factors from Tfr cells. After being cultured for 48 hours, the ratio of B cells (CD3−CD19+ cells) and CD38++ plasma cells (CD3−CD19−CD38+ cells) was detected using flow cytometry. ELISA experiments were used to detect Ig concentration.

### EDU Proliferation experiments

After MLC, B cells were sorted and were added with EDU staining reaction solution. After being cultured for 48 h, the culture medium was discarded, and the cells were washed with PBS for twice. 4% paraformaldehyde was added to fix these cells for 30 min, and then incubated with 2 mg/mL glycine on a decolorizing shaker for 5 min. After being washed with PBS for 3 times, then PBS with 0.5% TritonX-100 was added, and incubated on a decolorizing shaker for 10 min. Each group was added with EDU staining reaction solution, kept in a dark and dry place, and incubated at the room temperature on a decolorizing shaker for 30 min. After DAPI staining, the tluorescence was observed under the microscope.

### Statistical analysis

SPSS V16.0 was operated to statistically process the data, the quantitative data were indicated by (mean ± standard deviation). Two-sided Wilcoxon rank sum test was performed to compared the two-sample clinical data, Spearman rank method was used to measure the correlation and the experimental data were compared using t-test, p < 0.05 was considered with statistical significance.

## Electronic supplementary material

Supplementary information
