# Supplementary material for: Low proportion of follicular regulatory T cell in renal transplant patients with chronic antibody-mediated rejection
Source: Sci Rep. 2017 May 2;7:1322. doi: 10.1038/s41598-017-01625-3 (PMC5431051; doi:10.1038/s41598-017-01625-3)

**Low proportion of follicular regulatory T cell in renal transplant patients with chronic antibody-mediated rejection**

Wen Chen ^a^, Jian Bai ^a^, Haiyan Huang ^a^, Lili Bi ^a^, Xiangrui Kong ^a^, Yu Gao ^a^, Yong Han ^a^, Li Xiao ^a, †^, Bingyi Shi ^a, †^

a. Beijing Key Laboratory of Immunology Regulatory and Organ Transplantation, Basic Research Lab of Organ Transplant Institute, 309th Hospital of the People's Liberation Army, Beijing 100091, China

^†^ Corresponding author: Bingyi Shi, E-mail: shibingyi@medmail.com.cn;

Li Xiao, E-mail: xiaolilab309@163.com

Table S1. The baseline and clinical characteristics of recipients in renal transplantation

|  | Total (n=128) | CRAD (n=88) | Control (n=40) | *P* value |
| --- | --- | --- | --- | --- |
| Age (yr)  Male gender  BMI (Kg/m^2^)  Time after transplant (yr)  White blood cell  Lymphocyte  Tfh cell  Tfh1 cell  Tfh2 cell  Tfh17 cell  Tfr cell  Monocyte  Urea nitrogen  Creatinine (umol/L)  Uric acid  Total protein  GFR  Triglyceride  Total cholesterol | 42.8±11.2  42  25.2±4.9  4.77±1.75  7.9±2.8  27.0±8.7  1.25±0.75  0.32±0.05  0.39±0.055  0.33±0.03  0.13±0.016  0.60±0.25  7.46±4.0  116.4±49.9  377.3±80.5  67.8±10.8  68.3±27.8  1.80±0.9  4.60±1.3 | 41.7±10.7  28  25.7±5.3  4.86±1.73  7.8±3.3  26.7±8.2  1.28±0.78  0.4±0.07  0.39±0.057  0.36±0.034  0.11±0.013  0.61±0.3  8.11±4.5  235.3±48  396.6±73  68.5±10.6  56.9±16.2  1.77±0.95  4.77±1.4 | 45.4±12.3  14  24.8±4.6  4.5±1.58  8.0±1.3  27.6±7.8  1.17±0.67  0.57±0.02  0.31±0.05  0.27±0.03  0.17±0.023  0.58±0.22  5.8±1.4  73.2±15.8  333±83  66±11.6  94.7±31.6  1.85±0.8  4.2±0.9 | 0.428  0.628  0.187  0.208  0.814  0.765  0.481  ＜0.01  ＜0.01  0.023  ＜0.01  0.736  0.034  ＜0.01  0.053  0.574  0.004  0.823  0.190 |

Table S2. Correlation between Tfh cells, Tfh subsets, DSA and creatinine value

| Factors | DSA | Creatinine value |
| --- | --- | --- |
| Tfh cell  Tfh2 and Tfh17  Tfr cell | 0.1596 (P=0.3911)  0.6124 (P=0.0003)  -0.5090 (P=0.0035) | -0.01916 (P=0.9185)  0.2359 (P=0.1246)  -0.2311 (P=0.211) |

Figure S1 Effects of anti-rejection drugs on CTLA-4 expression and IL-21 secretion of Tfh cells. (a) The lymphocytes were stained with CD4, CXCR5 and CTLA-4; (b) Cyclosporine and tacrolimus promoted CTLA-4 expression compared with control group; (c) Sirolimus could enhance IL-21 secretion of Tfh cells. *p<0.05 (n=6) *versus* Control. Values are mean ± SD.


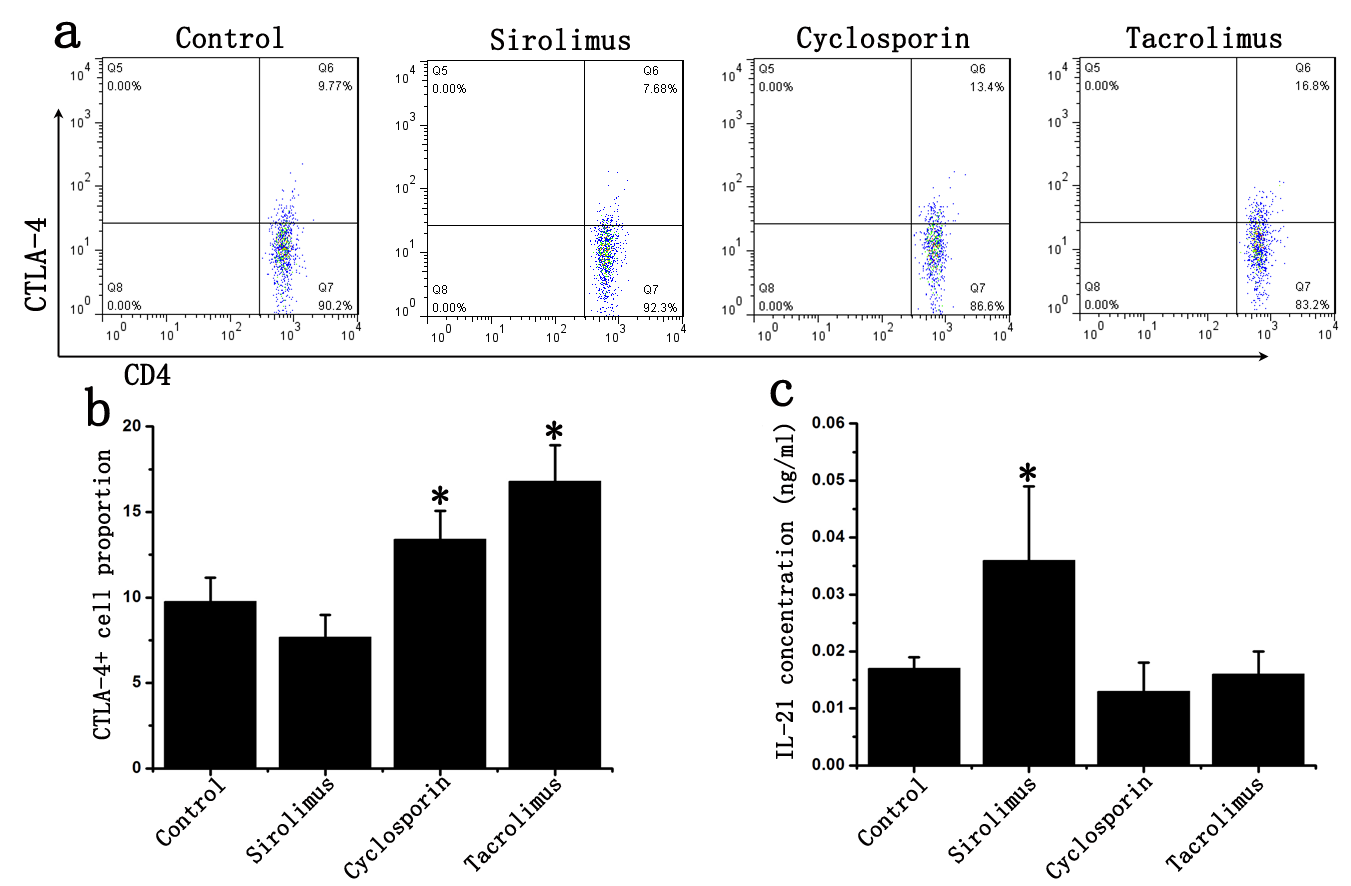


Figure S2. Effects of anti-rejection drugs on B cell proliferation. (a) Tfh cells were sorted from each group and then used for mixed lymphocyte culture. (b) Tfh cells in sirolimus, cyclosporine and tacrolimus group could not inhibit B cell proliferation.


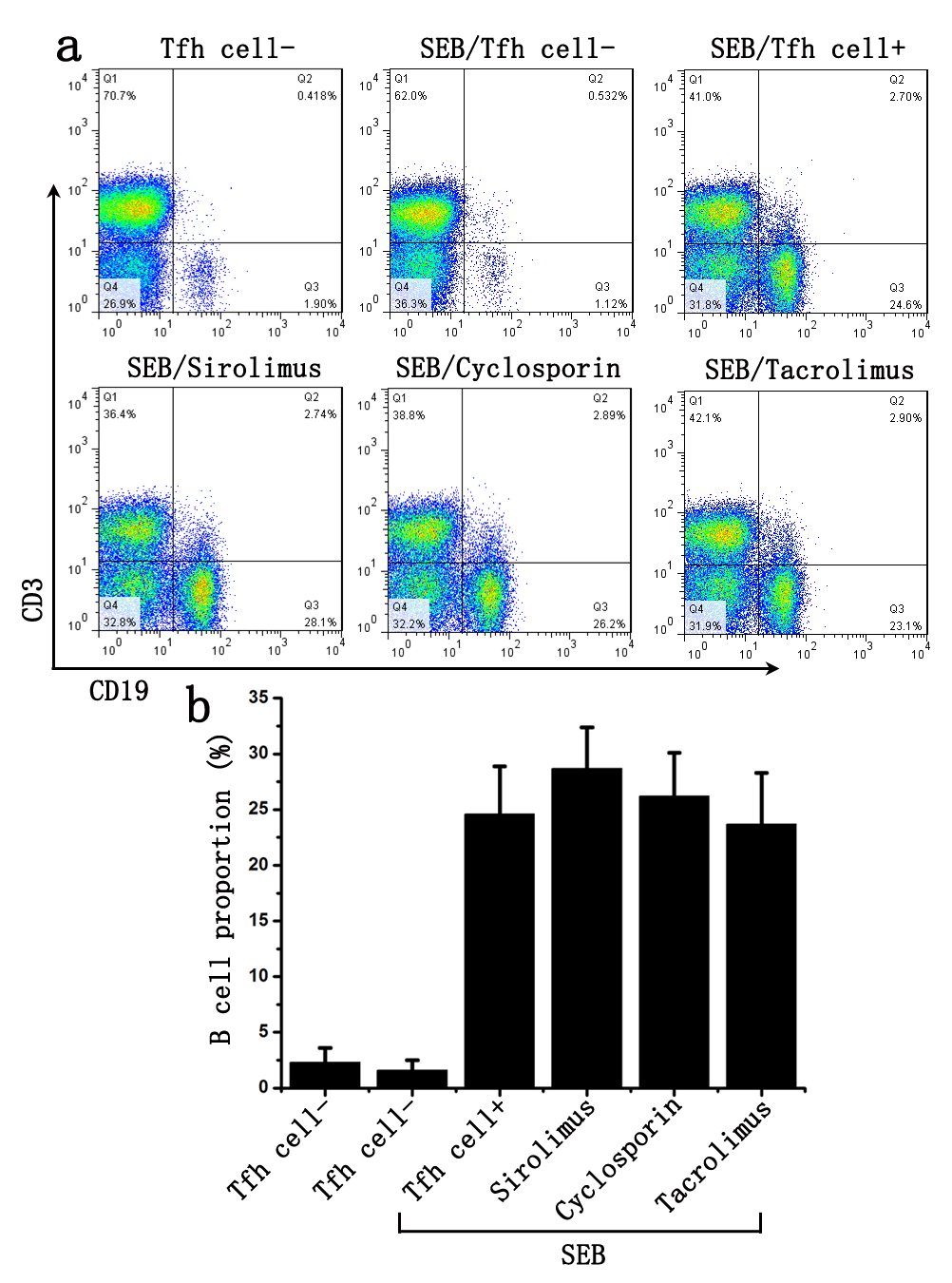


Figure S3. Tfr cells inhibited the IgG and IgA production from plasma cells, but had no significant effect on IgM production.


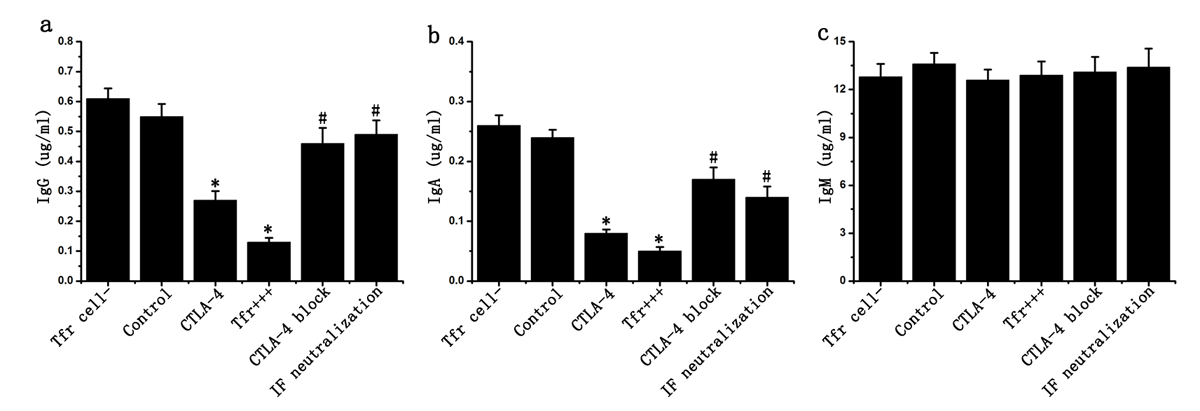


Figure S4. Tfr cells in AMR patients exerted normal inhibitory function. Tfr cells were sorted from each group and divided into many groups: Tfr cells normal ratio group (Control, a), Tfr cell from control patients (b), Tfr cell from Non-AMR patients (c) and Tfr cell from AMR patients (d). (e, f) Tfr cells isolated from AMR patients exerted the similar inhibitory effect on B cell proliferation and differentiation into plasma cells. *p<0.05 (n=6) *versus* Control. Values are mean ± SD.


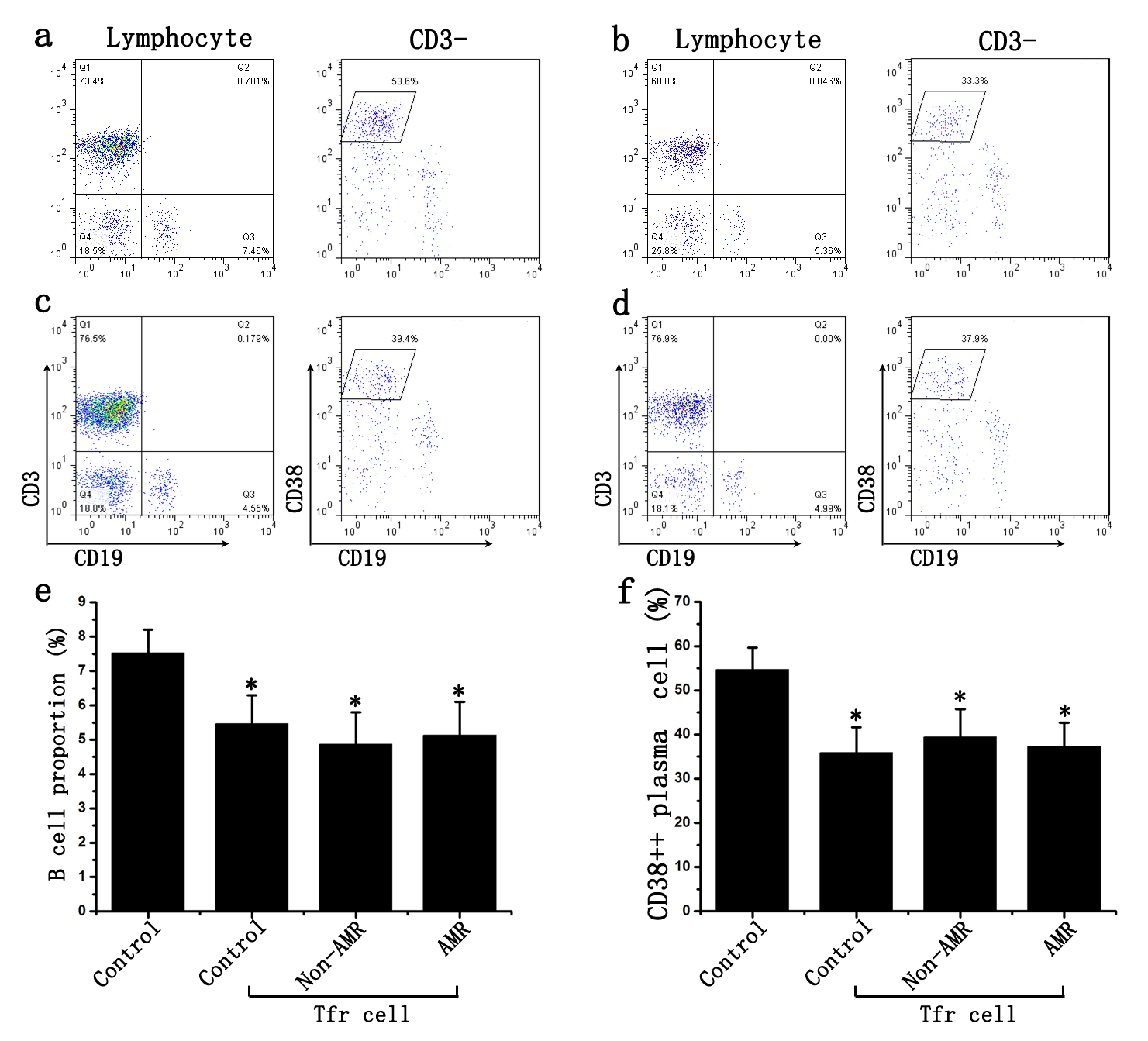

Supplement: Supplementary file 1 — Supplementary information [file 41598_2017_1625_MOESM1_ESM.doc]
